# RIBOSS detects novel translational events by combining long- and short-read transcriptome and translatome profiling

**DOI:** 10.1093/bib/bbaf164

**Published:** 2025-04-13

**Authors:** Chun Shen Lim, Alexandra K Gibbon, Anh Thu Tran Nguyen, Gabrielle S W Chieng, Chris M Brown

**Affiliations:** Department of Biochemistry, School of Biomedical Sciences, University of Otago, 710 Cumberland Street, Dunedin North, Dunedin 9016, New Zealand; Genetics Otago, University of Otago, 710 Cumberland Street, Dunedin North, Dunedin 9016, New Zealand; Department of Biochemistry, School of Biomedical Sciences, University of Otago, 710 Cumberland Street, Dunedin North, Dunedin 9016, New Zealand; Genetics Otago, University of Otago, 710 Cumberland Street, Dunedin North, Dunedin 9016, New Zealand; Department of Biochemistry, School of Biomedical Sciences, University of Otago, 710 Cumberland Street, Dunedin North, Dunedin 9016, New Zealand; Genetics Otago, University of Otago, 710 Cumberland Street, Dunedin North, Dunedin 9016, New Zealand; Department of Biochemistry, School of Biomedical Sciences, University of Otago, 710 Cumberland Street, Dunedin North, Dunedin 9016, New Zealand; Genetics Otago, University of Otago, 710 Cumberland Street, Dunedin North, Dunedin 9016, New Zealand; Department of Biochemistry, School of Biomedical Sciences, University of Otago, 710 Cumberland Street, Dunedin North, Dunedin 9016, New Zealand; Genetics Otago, University of Otago, 710 Cumberland Street, Dunedin North, Dunedin 9016, New Zealand

**Keywords:** ribosome profiling analysis method, gene annotation, Nanopore long-read direct RNA sequencing, transcriptome assembly, protein synthesis

## Abstract

Ribosome profiling is a high-throughput sequencing technique that captures the positions of translating ribosomes on RNAs. Recent advancements in ribosome profiling include achieving highly phased ribosome footprints for plant translatomes and more recently for bacterial translatomes. This substantially increases the specificity of detecting open reading frames (ORFs) that can be translated, such as small ORFs located upstream and downstream of the annotated ORFs. However, most genomes (e.g. bacterial genomes) lack the annotations for the transcription start and termination sites. This hinders the systematic discovery of novel ORFs in the ‘untranslated’ regions in ribosome profiling data. Here, we develop a new computational pipeline called RIBOSS to discover noncanonical ORFs and assess their translational potential against annotated ORFs. The RIBOSS Python modules are versatile, and we use them to analyse both prokaryotic and eukaryotic data. We present a resulting list of noncanonical ORFs with high translational potential in *Homo sapiens*, *Arabidopsis thaliana*, and *Salmonella enterica*. We further illustrate RIBOSS utility when studying organisms with incomplete transcriptome annotations. We leverage long-read and short-read data for reference-guided transcriptome assembly and highly phased ribosome profiling data for detecting novel translational events in the assembled transcriptome for *S. enterica*. In sum, RIBOSS is the first integrated computational pipeline for noncanonical ORF detection and translational potential assessment that incorporates long- and short-read sequencing technologies to investigate translation. RIBOSS is freely available at https://github.com/lcscs12345/riboss.

## Introduction

Ribosome profiling is a powerful technique for mapping the positions of ribosomes on RNAs, providing a snapshot of actively translated open reading frames (ORFs) [1, 2]. ORFs are regions of a nucleic acid sequence that have no stop codons. Ribosome profiling is routinely used for discovering actively translated ORFs for several key reasons. As ribosomes progress along the mRNA codon by codon, they generate a characteristic triplet periodicity profile in the footprint data [1, 2]. Triplet periodicity can be used to determine the correct reading frame for the translated ORFs and distinguish true translation events from background noise. Moreover, the density and distribution of ribosome footprints reveal the frequency and speed of translation occurring within an ORF. Therefore, the consistent footprint periodicity of an ORF provides direct evidence of active translation.

**Figure 1:**
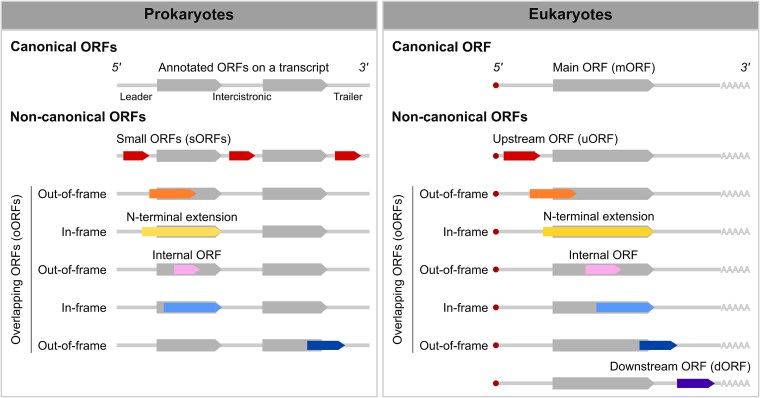
Types of open reading frames (ORFs) in prokaryotes and eukaryotes. ORFs can be divided into two main categories: primary ORFs (annotated and main ORFs for prokaryotes and eukaryotes, respectively) and noncanonical small ORFs (sORFs). These sORFs are 300-nt or shorter and can be categorized into two subtypes: Overlapping and nonoverlapping. In prokaryotes, nonoverlapping ORFs are sORFs with start and stop codons confined in the transcript’s 5′ leader, intercistronic, and 3′ trailer regions. Overlapping ORFs (oORFs) are those that overlap with annotated ORFs. This includes ORFs that result in N-terminal extensions of the annotated protein, as well as those entirely contained within annotated ORFs (sometimes referred to as internal ORFs). In eukaryotes, nonoverlapping ORFs include both upstream ORFs (uORFs) and downstream ORFs (dORFs) that are found in the 5′ leader and 3′ trailer sequences, respectively. Eukaryotic oORFs encompass both internal out-of-frame sORFs as well as in-frame and out-of-frame sORFs that overlap either the start or end of the main ORF. RIBOSS detects these noncanonical ORFs except for in-frame and internal ORFs.

Ribosome profiling allows the identification of various types of ORFs, including those corresponding to canonical protein-coding sequences (CDSs) (Fig. 1). In eukaryotes, these CDS regions can be called the main ORF (mORF) [3, 4]. While the term mORF is primarily associated with eukaryotes, the concept of a primary protein-coding sequence is relevant across all organisms. In prokaryotes, we will refer to these primary ORFs as the annotated ORFs [5, 6]. This difference in terminology reflects the distinct genetic architectures of prokaryotes and eukaryotes.

Ribosome profiling also reveals small ORFs (sORFs; 300 nt or shorter), frequently found in the leader or trailer sequences [5′ or 3′ untranslated regions (UTRs)] [5, 7–15] (Fig. 1). In eukaryotes, these sORFs are commonly called upstream ORFs (uORFs) or downstream ORFs (dORFs) [11]. Furthermore, ribosome profiling identifies overlapping ORFs (oORFs), which can be located entirely within the CDS (internal ORFs) or extended beyond the canonical coding region [11, 16]. Translation of these noncanonical ORFs highlights the complexity of translational landscapes.

Standard ribosome profiling involves RNase digestion of ribosome-protected mRNA fragments, followed by sucrose gradient fractionation, and RNA sequencing (RNA-seq). Significant advancements, such as ‘super-resolution’ ribosome profiling, particularly in plants, enabling more precise mapping of ribosome positions [12, 17]. Recently, ORFs discovered through ribosome profiling have been curated and released as a catalogue for the GENCODE human annotation project [7–9], highlighting the increasing recognition of noncanonical ORFs. Ribosome profiling has become indispensable for investigating gene expression and translational control, improving our understanding of fundamental cellular processes.

Despite these advances, complete gene annotation in nonmodel organisms remains a significant challenge. This is particularly true for bacteria, where high-quality, phased ribosome profiling data have only recently become available [18]. While this opens exciting new avenues for discovering novel bacterial translation events, the accurate identification of transcription start and termination sites, as well as intercistronic regions, is often lacking, even in important pathogenic bacteria. These regions are crucial for identifying and characterizing noncanonical translation events, which are increasingly recognized as important in transcriptional and translational regulation, essential bacterial physiology, and infection biology [5, 13, 19–23]. Similarly, in eukaryotes, sORFs located in the regions flanking the mORFs (uORFs and dORFs) are known to play key regulatory roles [14, 24–26]. These sORFs can act as *cis*-regulatory elements, affecting the translation of mORFs through mechanisms such as ribosome stalling, translational attenuation, or direct interactions with regulatory proteins. This regulatory interplay emphasizes the importance of comparing the translational potential of sORFs relative to their cognate mORFs, as such comparisons help determine the functional impact of these noncanonical ORFs. The broad functional roles of sORF-encoded peptides, from cellular physiology to development [27, 28], highlight the need for tools that go beyond the annotated regions to explore their important biological roles.

However, current ribosome profiling analysis tools focus on identifying all potential ORFs but lack the ability to quantitatively assess their relative translational potential and sequence homology to infer regulatory roles. This limitation hinders the comprehensive exploration of translational landscapes and the discovery of regulatory mechanisms mediated by noncanonical ORFs. To overcome these limitations, we have developed RIBOSS, a novel computational pipeline that integrates long- and short-read RNA sequencing data for reference-guided transcriptome assembly with ribosome profiling data to identify and characterize novel translational events beyond annotated regions. RIBOSS leverages state-of-the-art software for read alignment, transcriptome assembly, and ribosome footprint quantification. The goal is to accurately identify and assess the regulatory potential of sORFs, which is essential for uncovering the complex translational landscapes of diverse organisms.

RIBOSS has six dedicated modules for systematic ribosome footprint analysis, noncanonical ORF detection, and translational potential assessment. Unlike existing tools that primarily focus on ORF identification [6, 29, 30], RIBOSS distinctly compares the translational potential of noncanonical ORFs to that of the nearest mORF using statistical methods (Fig. 1, coloured versus grey). This comparison eliminates the need for species-specific training. Moreover, this approach allows for a nuanced understanding of the potential functional and regulatory roles of these noncanonical ORFs. Thus, RIBOSS offers an innovative approach to ribosome profiling data analysis, enabling researchers to explore the complex translational landscape of diverse organisms, including those where genome annotation is incomplete.

In this study, we demonstrate the utility of RIBOSS by applying it to three diverse datasets: the prokaryote *Salmonella enterica* serovar Typhimurium and the eukaryotic organisms *Homo sapiens* and *Arabidopsis thaliana*. While the identified novel translational events require experimental validation, our findings demonstrate that RIBOSS is capable of identifying such unusual events, providing new insights into the complexity of translational regulation in both prokaryotic and eukaryotic systems.

## Results and discussion

### Overview of RIBOSS modules

RIBOSS comprises six modules for ribosome footprint data analysis, including statistical comparison of translational potential between noncanonical and annotated ORFs (Fig. 1). The modules integrate established bioinformatics tools to allow ribosome profiling data analysis through an interactive computational environment, such as JupyterLab and Visual Studio Code.

The pipeline begins with optional genome-guided transcriptome assembly to leverage long-read sequencing data for organisms lacking comprehensive annotations (Fig. 1). The first step maps RNA-seq data (long and short reads) to a reference genome using minimap2 and bowtie2, respectively [31, 32]. Subsequently, transcriptome_assembly assembles transcriptomes from long and short reads using StringTie [33]. To take full advantage of RIBOSS, these optional steps are necessary for less-studied organisms lacking leader, trailer, or intercistronic sequences. Although transcriptome_assembly can utilize long-read alignments alone, we recommend combining both long and short reads to obtain highly accurate full-length transcripts [33]. These two initial steps prepare new transcriptomes for the RIBOSS core functions and can be skipped for well-annotated transcriptomes (see case studies for *H. sapiens* and *A. thaliana* data).

RIBOSS employs four core functions (operon_finder or ORF_finder, align_reads, and quantify_transcripts) to generate necessary data for downstream analysis. The operon_finder and ORF_finder functions predict possible operons and ORFs from the three forward reading frames of transcripts in prokaryotes and eukaryotes, respectively. These three forward reading frames are the inherent reading frames within any mRNA sequence, representing the three possible ways to group codons, each starting at a different nucleotide and forming consecutive triplets. These are the three frames considered during ORF prediction. By default, operon_finder and ORF_finder exclude annotated noncoding RNAs because the core statistical functions compare the translational potential between noncanonical and canonical ORFs. In addition, the in-frame ORFs and internal ORFs, including those in different isoforms, are excluded to avoid ambiguity.

The RIBOSS align_reads function maps ribosome profiling and RNA-seq short reads to the transcriptome using STAR [34]. STAR is a highly accurate aligner that utilizes suffix array indexing to enable efficient mapping. The quantify_transcripts function quantifies transcript isoform abundance using Salmon [35], which was chosen for bias mitigation and speed.

The outputs of ORF_finder/operon_finder and align_reads serve as inputs for the analyse_footprints function. This function evaluates triplet periodicity to assess the quality of the ribosome profiling data and predicts the footprint offset values for each ribosome footprint size. This step statistically selects high-quality footprints and correctly maps footprints to P-sites. The riboprofiler function then uses Ribomap [36] and the predicted offset values to assign footprints to protein-coding and noncoding regions at isoform resolution. These P-site-adjusted ribosome profiles are then used by the core RIBOSS analytical modules, which compare the translational potential between noncanonical and canonical ORFs [37, 38].

The main outputs include the blastp hits and Identical Protein Groups for noncanonical ORFs with significantly stronger translational potential than nearby canonical ORFs. Other output files include the assembled transcriptome, publication-ready footprint heatmaps and metagene plots, and the annotation tracks of the novel ORFs and P-site-adjusted ribosome profiles. These annotation tracks are viewable in common genome browsers, e.g. the University of California Santa Cruz (UCSC) Genome Browser, Integrative Genomics Viewer (IGV), or Artemis [39–41].

### RIBOSS combines long- and short-read data for transcriptome assembly with highly phased ribosome profiling data

Long-read RNA-seq is increasingly being used for bacterial transcriptome profiling, as it provides full-length transcriptome data that allow us to progress beyond traditional definitions of operons. Operons have classically been defined as a set of genes transcribed from a single promoter, often simplified as a block of genes in the genome [42]. While this operon definition is still widely used, it is primarily based on genetic organization and inferred transcription units. Long-read RNA-seq reveals a more nuanced picture of bacterial transcriptomes, showing that what appears to be a single operon based on genomic proximity may consist of multiple, distinct transcription units [43–46]. These transcription units, as defined by transcription start and termination sites, represent the actual functional units of transcription. For example, recent long-read sequencing studies have revealed complex transcriptional landscapes in bacteria, showing that what were previously considered single operons often contain multiple promoters and give rise to a variety of transcripts [43–46].

However, current prokaryotic annotation pipelines (e.g. National Center for Biotechnology Information Prokaryotic Genome Annotation Pipeline (NCBI PGAP) and Prokka) [47–49] still focus solely on primary protein-coding regions and noncoding RNAs. This hinders the discovery of novel translational events, including novel ORFs located in various regions of transcripts (Fig. 1). These elements, including transcription start/termination sites and intercistronic regions, are crucial for understanding biological functions [7]. RIBOSS addresses this limitation by uniquely combining long- and short-read RNA-seq data with highly phased ribosome profiling to analyse translation at noncanonical sites. The RIBOSS transcriptome_assembly function uses StringTie to generate high-quality, reference-guided transcriptomes. It integrates long-read coverage and short-read accuracy. This enables us to assess the relative translational potential between canonical and noncanonical ORFs to infer their regulatory roles. Thus, RIBOSS is useful for discovering novel translation events as long-read RNA-seq data become more prevalent for organisms with incomplete transcriptome annotations.

To showcase RIBOSS’s functionality in prokaryotes, we have analysed the first and only publicly available, highly phased *S. enterica* serovar Typhimurium ribosome profiling data [18]. However, as long-read RNA-seq data for this strain are not publicly available, we have assembled a new transcriptome to match the ribosome profiling data. Therefore, we supplemented our analysis using the only two publicly available long-read RNA-seq datasets from related *S. enterica* strains [50, 51]. These datasets also have limited sequencing depth. One of the datasets is a metatranscriptome of *S. enterica* serovar Enteritidis, *E. coli* O157:H7, and *Listeria monocytogenes*.

**Figure 2:**
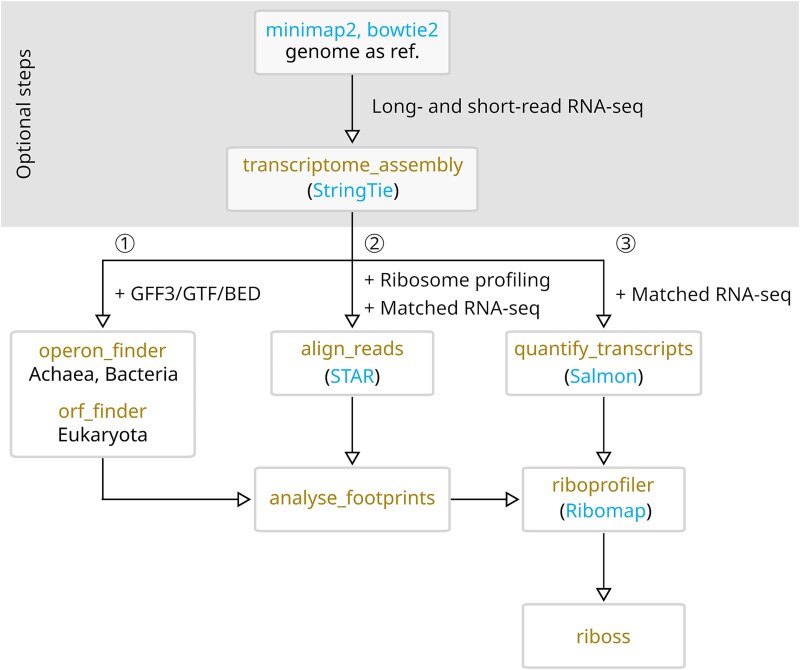
Ribosome profiling analysis workflow using RIBOSS. RIBOSS core functions integrate existing bioinformatics tools to provide interactive and modular functionality. The analysis starts with mapping long and short RNA reads using minimap2 and bowtie2, respectively [31, 32]. RIBOSS transcriptome_assembly function performs reference-guided assembly using StringTie [33], accepting either long-read alignments or combined long- and short-read alignments. Operon and ORF prediction: ① RIBOSS operon_finder predicts operons (prokaryotes) and ORF_finder predicts ORFs (eukaryotes) from the three forward frames of assembled transcripts. Read mapping and transcript quantification: ② RIBOSS align_reads maps ribosome footprints and RNA-seq data to transcript isoforms using STAR [34], ③ while quantify_transcripts estimates isoform abundance using Salmon [35], and riboprofiler assigns footprints by reading frame using Ribomap [36]. RIBOSS core analytical modules compare the translation of newly predicted ORFs with nearby annotated ORFs, enabling the identification of novel translated regions. RIBOSS also identifies blastp hits and identical protein groups [37, 38] for novel ORFs. Output files include the assembled transcriptome, publication-ready plots, and annotation tracks of significantly translated ORFs and P-site adjusted ribosome profile (viewable in common genome browsers, e.g. the UCSC Genome Browser, IGV, or Artemis [39–41]).

We have processed the two datasets separately, by mapping the long and short reads to *S. enterica* reference sequences or concatenated sequences of LT2, *E. coli* O157:H7, and *L. monocytogenes* using minimap2 [31] and bowtie2 [32], respectively (Fig. 2). To remove noise from other species, we have used reads mapped uniquely to LT2 chromosomes only. This approach has allowed us to leverage available long- and short-read data while focusing on the *S. enterica* genome and ORF annotation for reference-guided transcriptome assembly. This has also allowed us to obtain all annotated CDSs along with leader, trailer, and intercistronic regions supported by long- and short-read sequencing. However, this approach has limitations. As strain-specific long-read data are unavailable, our analysis cannot report transcription units specific to the *S. enterica* LT2 strain. While our goal is to demonstrate RIBOSS’s functionality in investigating novel translation events in organisms with incomplete transcriptome annotations, future studies with strain-specific long-read data will be necessary to validate these findings and provide a more comprehensive understanding of its translatome. As long-read RNA-seq technologies become more accessible and affordable, enabling the assembly of more complete transcriptomes, RIBOSS will be more valuable for discovering novel translational events.

### RIBOSS predicts open reading frames and operons in transcriptome assembly

The RIBOSS pipeline starts from the transcriptome_assembly function to assemble transcriptomes using Nanopore or PacBio long-read alignment files or a combination of long- and short-read alignment files. This function has presets for prokaryotic and eukaryotic transcriptome data. In particular, the preset for prokaryotes can suppress the assembly of spliced transcripts.

In this *S. enterica* example, we have combined Nanopore direct RNA-seq and Illumina short-read RNA-seq data to assemble a metatranscriptome guided by concatenated genomes and reference annotations of the three bacterial species [51]. We have also assembled a separate *S. enterica* transcriptome using a Nanopore complementary DNA (cDNA) sequencing data as mentioned above [50]. We have merged these assembled transcripts with the *S. enterica* reference gene annotation, generating a total of 4672 transcripts (Fig. 2). The median length of the transcripts is 861 nt.

We have used the RIBOSS operon_finder function to detect all possible ORFs in the three forward frames of the assembled transcripts and matched them with annotated ORFs (Fig. 2 ①). By default, operon_finder (for archaea and bacteria) and ORF_finder (for eukaryotes) exclude annotated noncoding RNAs from the analysis. These two functions also remove the ORFs in-frame with annotated ORFs to avoid false-positive results. As the ORFs found in a transcript isoform can overlap the annotated ORFs in a different isoform, the functions also remove them from the analysis. To achieve this, the functions convert the transcript level coordinates to the genomic level and compare them with the annotated ORFs.

**Figure 3:**
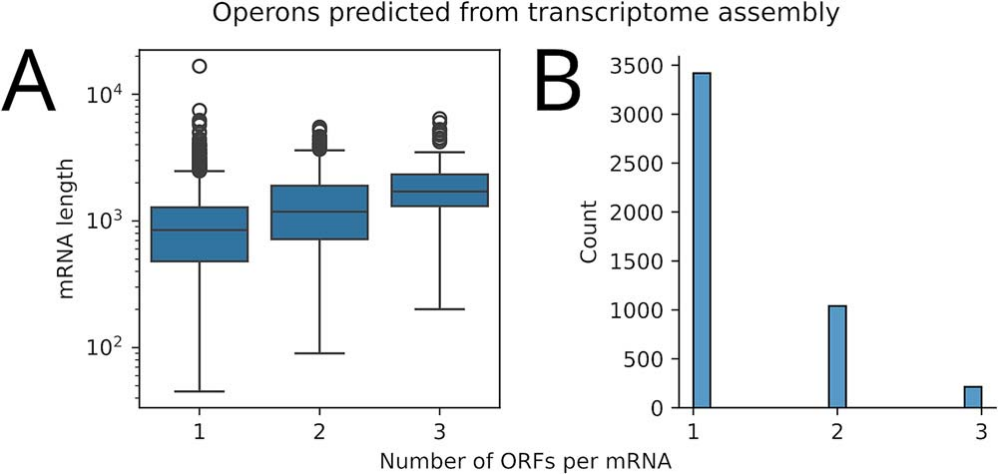
Operons detected through reference-guided transcriptome assembly. The RIBOSS transcriptome_assembly and operon_finder functions were used for this analysis. A total of 4681 transcripts were assembled by merging two independent transcriptome datasets with the reference gene annotation for *S. enterica* serovar Typhimurium LT2. The datasets include a hybrid of Nanopore long-read direct RNA-seq and Illumina short-read RNA-seq metatranscriptome data (a cocktail of *S. enterica* serovar Enteritidis, *Escherichia coli* O157:H7, and *Listeria monocytogenes*) and Nanopore cDNA sequencing data (*S. enterica*). The median length of the assembled transcripts is 929 nt (A), where 1253 of these transcripts harbour two or more annotated ORFs (B).

We have found 1.3 genes per operon (transcription unit) for *S. enterica* (Fig. 3), which is similar to 1.4 genes per operon in *E. coli* [52–54]. Notably, 1253 of these transcripts harbour two or more annotated ORFs, which is greater than the number of *E. coli* polycistronic operons (*N* = 788). This is expected as a more recent study using PacBio long-read iso-seq has also extended the *E. coli* operon annotation [44].

Moreover, we have defined the regions initially thought to be untranslated (e.g. the leaders, trailer, and intercistronic regions) and included these regions for ORF prediction. This approach can stimulate further investigation of these novel ORFs, e.g. their regulatory roles based on the operon model for RNA as a post-transcriptional circuit [42]. This enables users to make the most of ribosome profiling to investigate translational control in polycistronic transcripts assembled using a hybrid of long and short reads [52, 53].

### RIBOSS compares the triplet periodicity of the ribosome footprints aligned to the reading frames

**Figure 4:**
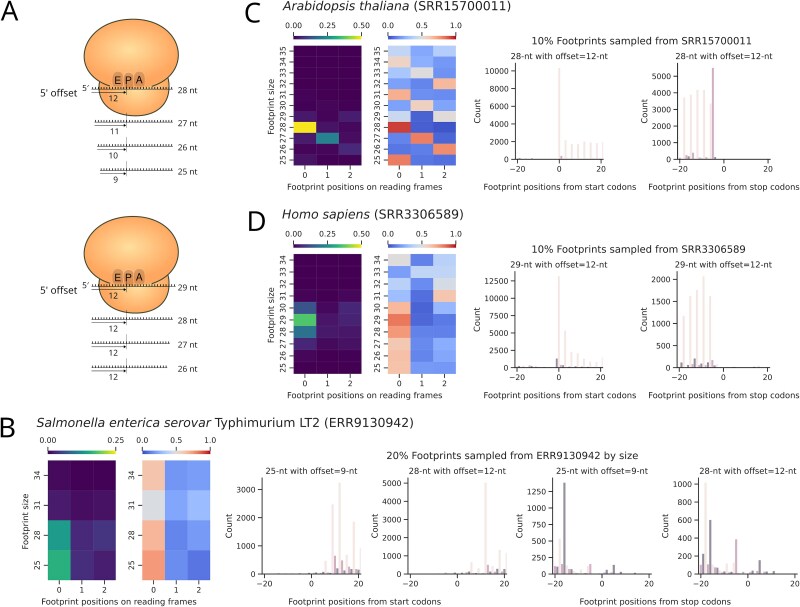
The RIBOSS analyse_footprints function evaluates ribosome footprint periodicity by size. (A) This function automatically determines P-site offsets by aligning footprint positions from the 5′ ends of reads. Users can optionally choose to offset footprint positions from the 3′ ends or specify an offset value for the footprint size of 28 nt. This value will be automatically extended to offset different footprint sizes. By default, the analyse_footprints function selects the most abundant footprint sizes for downstream analysis as these footprints typically display the strongest triplet periodicity. Users can invoke statistical analysis to assess triplet periodicity across different footprint sizes. This analysis allows for the automated selection of high-quality footprint sizes with statistically similar periodicity. In (B–D), the left heatmaps depict the absolute footprint abundance (range 0–0.5) by footprint size (range 25–35 nt) and reading frame (frames 0, 1, 2) after 5′ offset. The right heatmaps show the relative footprint abundance for each footprint size across all three reading frames; for each footprint size, the relative abundance across three frames sums to 1. The bar plots depict the signature high–low–low pattern of footprint periodicity around the start and stop codons. Users can see the offset values specific to different footprint sizes as the bar plot titles. For the *S. enterica* data (B), the 5′ offset method produced consistent triplet periodicity for the footprint sizes 25 and 28 nt. In contrast, the *A. thaliana* (C) and *H. sapiens* (D) data show a clear triplet periodicity for the footprint sizes 28 and 29 nt, respectively.

The RIBOSS align_reads function maps ribosome profiling and RNA-seq short reads to the transcriptome using STAR [17] (Fig. 2 ②). As ribosomes move along mRNA in codon increments, and canonical footprint sizes are around 25–29 nt, aligned footprint positions must be adjusted to the ribosomal P- or A-site (Fig. 4A). The analysed_footprints function automates footprint adjustment by calculating footprint positions relative to start and stop codons based on user-specified footprint sizes. Footprint positions are adjusted from the 5′ end, but 3′ adjustment is also an option. RIBOSS analysed_footprints generates heatmaps of aligned footprints by frame and metagene plots for selected footprint sizes (Fig. 4B–D).

Translated regions exhibit triplet periodicity, a key signature of translation elongation during protein synthesis. The analysed_footprints function uses the bootstrap G-test, *post hoc* tests, and/or odds ratio to evaluate triplet periodicity, enabling automated selection of high-quality footprints. High-quality footprints for *S. enterica* are 25 nt and 28 nt, with fewer 34-nt footprints (Fig. 4B). These footprints were generated using RNase I [18]. Although S7 MNase-treated footprints produce some degree of periodicity in *S. enterica*, we have decided not to use them because MNase preferentially cleaves upstream of A and T bases and may potentially produce artefacts [55].

In two further case studies, we demonstrate RIBOSS on well-annotated, highly complex eukaryotic genomes. We have used ribosome profiling data from the human cell line HeLa, which exhibits strong triplet periodicity as shown previously [56]. We have also used recently published, high-quality ‘super-resolution’ ribosome profiling data from *A. thaliana* Col-0 ecotype seedings [12] to illustrate RIBOSS’s versatility across eukaryotic species. High-quality footprints are 28-nt for *A. thaliana* (Fig. 4C) and 29-nt for *H. sapiens* (Fig. 4D), highlighting the importance of careful footprint selection for each dataset. The analysed_footprints function simplifies this process by generating heatmaps, metagene plots, and a text file containing selected footprint sizes and offset values, enabling easy visual inspection.

The selected footprint sizes and offset values from analysed_footprints are crucial for the riboprofiler function. This function uses Ribomap [36] to correctly assign footprints to coding and noncoding regions at isoform resolution. We have previously demonstrated that Ribomap can accurately assign eukaryotic ribosome footprints to transcript isoforms [57].

### RIBOSS reports statistically significant novel translational events

The core riboss function integrates upstream data and compares the translational potential of predicted ORFs and nearby annotated ORFs, i.e. prokaryotic annotated ORFs or eukaryotic mORFs (prokaryotic annotated ORFs or eukaryotic mORFs). As ribosome profiles along individual ORFs are often sparse (except for highly expressed proteins), riboss tallies footprints aligned by frame for each ORF. The function then (i) creates a 2 × 3 contingency table for each pair of predicted and neighbour-annotated ORFs (two ORFs × three frames), and (ii) compares triplet periodicity between paired ORFs using bootstrap G-tests and *post hoc* tests [58]. For each ORF pair, riboss calculates the odds of translation occurring on the predicted ORF using a 2 × 2 contingency table (two ORFs × footprints aligned to frames 0 and two other frames). Stringent statistical thresholds are used to identify novel ORFs (odds ratio > 1 and FDR-adjusted *P*-values <.05 for bootstrap G-tests and *post hoc* tests). In essence, riboss utilizes aggregated triplet periodicity to evaluate the possibility of predicted ORF being translated relative to its neighbour-annotated ORF. A predicted ORF meeting these statistical criteria is considered to have a greater translational potential relative to its paired annotated ORFs and is designated as the ‘boss’ of that transcript. If the *P*-values are significant before correction but not after, the translational potential of the paired ORFs is a ‘tie’. If the *P*-values are insignificant before correction, the annotated ORFs retain the ‘boss’ status.

**Figure 5:**
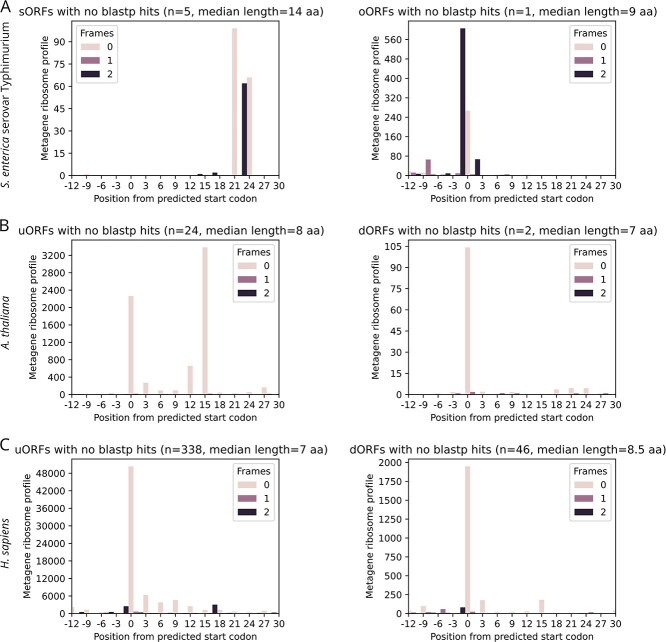
RIBOSS detects novel ORFs in prokaryotic and eukaryotic transcriptomes. RIBOSS compares the translational potential of predicted noncanonical ORFs with annotated ORFs and reports statistically significant results, along with metagene plots and blastp search results. (A) Metagene ribosome profiles for small ORFs (sORFs) and out-of-frame overlapping ORFs (oORFs) detected in the newly assembled *S. enterica* transcriptome. (B) Metagene ribosome profiles for upstream ORFs (uORFs) and downstream ORFs (dORFs) detected in the *A. thaliana* reference transcriptome. (C) Metagene ribosome profiles for uORFs and dORFs detected in the *H. sapiens* reference transcriptome. See also Fig. 6 for specific examples of these noncanonical ORFs.

**Figure 6:**
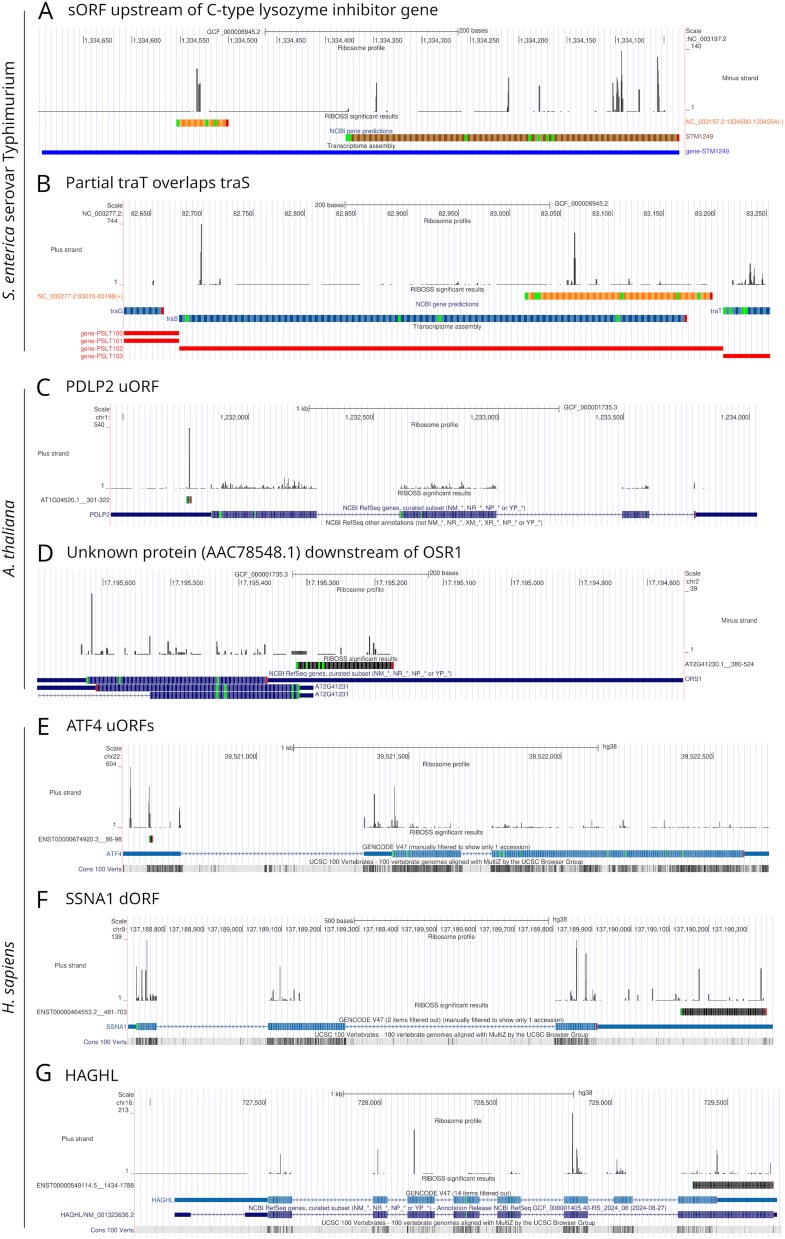
RIBOSS outputs visualized in the UCSC genome browser. RIBOSS generates genome-browser-compatible tracks in BED12, genePred, bigGenePred, and bedGraph formats. bigGenePred tracks display AUG start codons, stop codons, and amino acid translations for noncanonical ORFs in prokaryotes and eukaryotes. Other start codons may be used, especially in prokaryotes. (A) A novel sORF upstream of the C-type lysozyme inhibitor gene. (B) The partial plasmid entry exclusion gene *traT* overlapping *traS*. (C) A uORF of the PlasmoDesmata-Located Protein 2 (*PDLP2*) gene. (D) An uncharacterized ORF (AAC78548.1) downstream of the *OSR1* gene. (E) One of the well-characterized *ATF4* uORFs. (F) A dORF of the SS Nuclear Autoantigen 1 (*SSNA1*) gene. (G) An ORF downstream of the Hydroxyacylglutathione Hydrolase–Like gene (*HAGHL*), likely due to incomplete GENCODE annotation. For *A. thaliana* and *H. sapiens* examples, relevant reference transcript isoforms are shown to provide context.

In *S. enterica*, 10 predicted ORFs (~0.6% of possible sORFs and oORFs in the assembled transcripts) have passed the statistical thresholds. Six of these are novel, as they lack significant blastp hits (Fig. 5A). One example is an sORF upstream of the C-type lysozyme inhibitor gene (Fig. 6A). In *A. thaliana* and *H. sapiens*, 28 and 426 predicted ORFs have passed the thresholds, respectively. Most lack significant blastp hits (Fig. 5B and C), including the uORF and dORF of the PlasmoDesmata-Located Protein 2 (*PDLP2*) and SS Nuclear Autoantigen 1 (*SSNA1*) genes in *A. thaliana* and *H. sapiens*, respectively (Fig. 6C and F).

Among predicted ORFs with significant blastp hits, we have found a partial plasmid entry exclusion gene *traT* overlapping the annotated *traS* (Fig. 6B). Although this may be due to genome assembly errors in LT2 or other *S. enterica* strains, previous studies have observed a number of overlapping genes in prokaryotes [16, 59–64]. In *A. thaliana*, BLASTP has returned an uncharacterized ORF (AAC78548.1) located in the long 3′ UTR of Organ Size Related 1 (*OSR1*) (Fig. 6D). Notably, the 5′ end of this uncharacterized ORF overlaps the 5′ end of the opposite ORF, suggesting that they share a promoter region. Therefore, this uncharacterized ORF may be transcribed separately via a bidirectional promoter as an antisense gene. In *H. sapiens*, our analysis has recovered one of the well-characterized Activating Transcription Factor 4 (*ATF4*) uORF, validating RIBOSS’s functionality (Fig. 6E). A significant blastp hit for an ‘ORF’ downstream of the Hydroxyacylglutathione Hydrolase Like gene (*HAGHL*) likely indicates incomplete annotation in GENCODE, as this dORF is in-frame with the NCBI RefSeq annotated *HAGHL* mORF (Fig. 6G). We acknowledge that these novel ORFs warrant experimental validation, as they could be artefacts resulting from structured RNAs that were not completely digested during RNase treatment [65, 66]. Still, our results demonstrate the potential of RIBOSS to reveal unusual translational events and highlight areas where current annotations may be incomplete or require refinement.

### RIBOSS demonstrates superior precision and recall in noncanonical open reading frame detection

Direct comparison with other ribosome-profiling-based ORF prediction tools like ribotaper [29] and ribotricer [30] is challenging for two key reasons. Firstly, RIBOSS uses a unique comparative approach to assess the translational potential of noncanonical ORFs relative to their nearest annotated ORFs. In contrast, existing tools aim to return all potential translating/translatable ORFs *de novo*. These fundamental differences in methodology and purpose limit direct comparison. Secondly, benchmarking is hindered by the lack of a definitive ‘gold-standard’ dataset of validated noncanonical ORFs. Although we have used carefully curated datasets for benchmarking, the low number of noncanonical ORFs annotated and missing/insufficient ribosome footprint coverage remain limitations. While *de novo* tools predict all potential ORFs, RIBOSS focuses on discovering noncanonical ORFs by leveraging existing ORF annotations. This comparative strategy reduces false-positive detections (Supplementary Tables S1–S4 and Table 1) and enables users to infer the potential regulatory roles of predicted ORFs. Therefore, RIBOSS complements existing *de novo* ORF prediction tools and gene prediction pipelines by focusing particularly on noncanonical ORFs.

**Table 1:** Performance scores of ribosome profiling analysis tools on eukaryotic data.

Datasets	Tools	Performance scores			
		AUROC	AUPRC	MCC	F1
*A. thaliana*	RIBOSS	0.76	**0.51**	**0.72**	**0.68**
	Ribotricer	**0.86**	0.07	0.27	0.17
*H. sapiens*	RIBOSS	0.65	**0.26**	**0.48**	**0.44**
	Ribotricer	**0.70**	0.15	0.35	0.37

Note: The best results are marked in bold. AUROC, area under the receiver operating characteristic curve; AUPRC, area under the precision–recall curve; MCC, Matthews correlation coefficient; F1, harmonic mean of precision and recall.

To compare RIBOSS to ribotricer, the state-of-the-art tool designed for analysing eukaryotic ribosome profiling, we have established positive and negative control sets using a carefully designed methodology inspired by the ribotricer paper. To ensure a fair comparison, we have identified a common set of candidate ORFs by taking the intersection of ORFs predicted by both RIBOSS orf_finder and ribotricer prepare-orfs. This is a crucial step to ensure that both tools can be evaluated on the same set of ORFs. From this intersected set, we have defined our positive and negative controls. For positive controls, we have tested the ability of the tools to correctly classify noncanonical ORFs as translating using ribosome profiling data. These noncanonical ORFs are *A. thaliana* uORFs from the Araport11 annotation and Ribo-Seq ORFs, representing noncanonical ORFs in *H. sapiens* with expressed peptides found in proteomics data and recently incorporated into the GENCODE human annotation project [7–9]. For negative controls, we have used RNA-seq data to test the ability of the tools to correctly classify all the intersected sets of ORFs as nontranslating. Particularly, we have used previously published, high-quality ribosome profiling datasets for *A. thaliana* and HeLa cells, along with matched RNA-seq datasets (as described above). We have retained only ORFs with at least 10 footprint counts. Through this whole process, we have generated 78 uORFs (positives) and 97 754 ORFs (negatives) for *A. thaliana* and 126 Ribo-Seq ORFs (positives) and 4475 ORFs (negatives) for *H. sapiens*. Thus, the benchmark compares the tools’ ability to correctly identify ORFs from the ribosome profiling data (as true positives), while avoiding the ORFs being predicted from the RNA-seq data (as true negatives), all within the context of the initial, intersected ORF set. Any ORFs identified from RNA-seq would count as false positives.

We have retrained ribotricer on the *A. thaliana* and *H. sapiens* ribosome profiling datasets to optimize its phase-score cutoffs. This is an optional step and has improved ribotricer’s performance scores, e.g. F1 scores by 0.15 and 0.08 in *A. thaliana* and *H. sapiens*, respectively. Although RIBOSS requires no data-specific training, it predicts fewer false positives than ribotricer and demonstrates higher overall performance [Supplementary Tables S1–S4 and Table 1, higher area under the precision–recall curve, Matthews correlation coefficient (MCC), and F1 scores]. In contrast, ribotricer predicts more true positives, as indicated by higher area under the receiver operating characteristic curve scores. Thus, within the limitations of our benchmarking approach and sample size, RIBOSS demonstrates superior precision and recall in noncanonical ORF detection.

Ribotricer focuses on *de novo* ORF prediction with fewer functionalities and input parameters, which may be advantageous for users seeking this specific task. In contrast, RIBOSS is designed as an end-to-end solution for more comprehensive analysis and greater downstream utility. Ribotricer’s output formats (except for wiggle) may require additional processing for compatibility with standard bioinformatics tools. In contrast, RIBOSS generates output files in standard formats (BED12, genePred, bigGenePred, and bedGraph) directly compatible with widely used tools like BEDTools, UCSC Genome Browser, IGV, and Artemis [39–41, 67]. The bigGenePred files provide user-friendly visualization of ORF features (start/stop codons, amino acid translations), while bedGraph files enable ribosome profile visualization. Furthermore, RIBOSS provides a clear, step-by-step workflow through an interactive computational environment, such as JupyterLab and Visual Studio Code.

In summary, RIBOSS is a new Python package that can identify novel translational events. RIBOSS integrates diverse data types to provide a more comprehensive understanding of the translational landscapes. RIBOSS has demonstrated versatility in analysing ORFs’ translational potential in both eukaryotes and prokaryotes.

## Methods

### Data

The ribosome profiling and matched RNA-seq data for *S. enterica* serovar Typhimurium strain LT2 were retrieved from PRJEB51486 [18]. This translatome data corresponds to LT2 cells harvested at OD600 of 1, i.e. during the peak production of the SPI-1 transcriptional master regulator HilA [18]. Nanopore long-read direct RNA-seq and Illumina short-read RNA-seq data for a cocktail of *S. enterica* serovar Enteritidis (ATCC 13076), *E. coli* O157:H7 (ATCC 43895), and *L. monocytogenes* (ATCC 19115) were retrieved from PRJNA609733 [51]. This metatranscriptome data corresponds to bacteria grown in brain heart infusion media and romaine lettuce (*Lactuca sativa* L. var. longifolia) juice extract at 37°C for 24 h. An independent Nanopore long-read cDNA sequencing data for *S. enterica* was retrieved from SRX20554650 [50]. The reference genomes and General Feature Format (GFF) annotation files were downloaded from NCBI RefSeq [68].

The ribosome profiling and matched RNA-seq data for *A. thaliana* Col-0 ecotype seedings and *H. sapiens* were retrieved from PRJNA759858 [12] and PRJNA316618 [56], respectively. The reference genomes and Gene Transfer Format (GTF) annotation files for *A. thaliana* (TAIR10) and *H. sapiens* (hg38) were retrieved from the UCSC Genome Browser, GENCODE (v47 release), and TAIR [39, 69, 70]. Human Phase II Ribo-Seq ORFs (preprint’s supplementary data); Araport11 uORFs (October 2023 release; TAIR10 database) were used as positive sets for benchmarking [8, 69, 71].

### Sequence alignment

For *S. enterica*, long and short reads were aligned to the LT2 strain reference sequences (chromosome NC_003197.2 and plasmid pSLT NC_003277.2) or concatenated sequences of LT2, *E. coli* O157:H7 (chromosome NZ_CP008957.1 and plasmid NZ_CP008958.1), and *L. monocytogenes* (NC_017544.1) using minimap v2.28 [31] and bowtie2 [32], respectively.

The ribosome profiling and matched RNA-seq reads were aligned to the newly assembled *S. enterica* transcriptome or *A. thaliana*/*H. sapiens* reference transcriptomes using build_start_index and align_reads employing STAR v2.7.11b [34]. The build_start_index and align_reads functions are part of the wrapper.py module. SAMTools v1.21 were used to merge alignment files and convert the file formats [72].

### Transcriptome assembly

Reference-guided transcriptome assembly was performed using a hybrid of Nanopore long-read and/or Illumina short-read alignment files. Specifically, two separate transcriptomes were assembled, each using one of the two available long-read RNA-seq datasets from related *S. enterica* strains. Both assemblies were guided by the *S. enterica* reference genome and annotations. This was handled by transcriptome_assembly which employs StringTie v2.2.3 for transcriptome assembly [33], the UCSC Genome Browser’s KentUtils for file format conversion [39], and BEDTools v2.31.1 for transcript sequence extraction [67]. This step allowed us to obtain all annotated CDSs along with leader, trailer, and intercistronic sequences supported by long- and short-read sequencing (with caveats noted). The RIBOSS transcriptome_assembly function has a preset for prokaryotes to avoid spurious isoforms and spliced transcripts. The fraction of predicted transcript isoforms used is at least 0.1 of the most abundant transcript (default: 0.01). The minimum junction coverage used is 1000 for prokaryotes (to prevent prediction of spurious spliced transcripts) and 1 for eukaryotes (to include splicing) as default. The transcriptome_assembly function is part of the wrapper.py module. Finally, the two newly assembled transcriptomes were merged with the reference annotations to create a comprehensive transcriptome for downstream analysis

### Open reading frame and operon detection

The operon_finder and orf_finder functions detect ORFs in the three forward frames of prokaryotic and eukaryotic transcripts, respectively. These include annotated ORFs and out-of-frame oORFs for prokaryotes and eukaryotes, sORFs for prokaryotes, and upstream and downstream ORFs for eukaryotes. To classify ORFs as such, PyRanges v0.1.2 was used to join the detected ORFs on genomic location [73]. Unlike operon_finder, orf_finder was designed to handle the coordinates of splice junctions in eukaryotes. These functions are part of the orfs.py module.

### Ribosome footprint analysis

The triplet periodicity of ribosome footprints was examined using analyse_footprint. This includes subsampling the ribosome profiling alignment files using pysam v0.22.1 [74] to reduce data volume for efficient memory usage and faster plotting. The footprint positions were adjusted to P-site programmatically (Fig. 4).

### Transcript quantification and ribosome footprint assignment

Transcript levels were determined by quantify_transcripts employing Salmon v1.10.3 [35]. The ribosome footprints were assigned to reading frames and other ‘untranslated’ regions of transcripts using the C++ executable riboprof in Ribomap v1.2 [36].

### Comparison of translation potential between open reading frames

The Ribomap output from riboprofiler was used to calculate the footprint counts by reading frame for each ORF. The ribosome footprint profile of each codon is denoted as a row vector:


(1) \begin{equation*} {w}_i=\left[{f}_{i0}\\{f}_{i1}\ \ {f}_{i2} \right] \end{equation*}

where ${f}_{i0}$, ${f}_{i1}$, and ${f}_{i2}$ represent the footprint counts in reading frames 0, 1, and 2 for the $i$-th codon, respectively; $i$ represents the codon position in the ORF, from 1 to $N$; $N$ is the total number of codons in an ORF; and $f=0,1,2$.

For each ORF, the total ribosome footprint profile (sum of profiles across all codons) is denoted as $W$:


(2) \begin{equation*} W=\sum_{i=1}^N{w}_i=\sum_{i=1}^N\left[{f}_{i0}\ {f}_{i1}\ {f}_{i2}\ \right]=\left[\sum_{i=1}^N{f}_{i0}\ \sum_{i=1}^N{f}_{i1}\ \sum_{i=1}^N{f}_{i2}\ \right] \end{equation*}

If ${\sum}_{i=1}^N{f}_{i0}<{\sum}_{i=1}^N{f}_{i1}+{\sum}_{i=1}^N{f}_{i2}$, the ORF is classified as ‘no periodicity’ and no further analysis will be performed. Otherwise, the translational potential of a given pair of noncanonical ORF and annotated ORF will be compared.

The corresponding row vectors $W$ for a pair of noncanonical ORF and annotated ORF were tabulated as a 2 × 3 contingency table. Bootstrap G-tests were performed on these tables using an approach adopted from resamp v1.7.4 [75]. *Post hoc* tests were performed as a previously published method [76] using SciPy v1.14.1 [77]. As G-tests are inherently two-sided tests, odds ratios were calculated to determine the directionality of the results. *P*-value correction was done using statsmodels [78].

### Sequence homology searching

The main RIBOSS function includes sequence homology search for significantly translated ORFs using the BLASTP module of Biopython [38, 79]. The postprocessing steps include parsing the blastp results using the topiary read.py module [80] and retrieving the Identical Protein Groups of the blastp hits using efetch in the NCBI Entrez module of Biopython [37].

### Data processing and visualization

Pandas v2.2.3 and NumPy v1.26.4 were used for data processing [81–83]. Plots were made using seaborn v0.13.2 and Matplotlib v3.9.2 [84, 85]. The UCSC Genome Browser [39] was used to visualize the tracks generated by RIBOSS. The tracks include transcriptome assembly, ribosome profiles, and predicted ORFs in BED, bedGraph, and bigGenePred formats, respectively.

**Key Points**

- RIBOSS solves gene annotation issues by reference-guided transcriptome assembly using long- and short-read RNA-seq data.
- RIBOSS discovers potential novel translational events in the newly assembled transcripts.
- RIBOSS modules can be used to analyse eukaryotic or prokaryotic datasets.

## Supplementary Material

RIBOSS_SM_bbaf164

## Data Availability

RIBOSS is a Python package consisting of six modules. The source code is freely available on GitHub (https://github.com/lcscs12345/riboss). This package and its dependencies can be rapidly installed through the conda environment file using Miniforge3 v24.7.1-2 [86]. The Jupyter notebooks and the results are available on GitHub (https://github.com/lcscs12345/riboss_paper). Raw sequencing datasets for *S. enterica* are available on NCBI BioProject/SRA (PRJNA609733 and SRX20554650) and ENA (PRJEB51486). Raw ribosome profiling and matched RNA-seq data for *A. thaliana* and HeLa cells are available on PRJNA759858 and PRJNA316618, respectively.

## Notes

Biographical note: RIBOSS is a pipeline solving gene annotation problems using reference-guided transcriptome assembly and ribosome profiling.
