# Supplementary material for: RIBOSS detects novel translational events by combining long- and short-read transcriptome and translatome profiling
Source: Brief Bioinform. 2025 Apr 13;26(2):bbaf164. doi: 10.1093/bib/bbaf164 (PMC11994033; doi:10.1093/bib/bbaf164)
Supplement: RIBOSS_SM_bbaf164 [file riboss_sm_bbaf164.docx]

**SUPPLEMENTARY MATERIALS**

**Table S1. Prediction outcomes for RIBOSS on *A. thaliana*.**

|  |  | Predicted outcomes | |
| --- | --- | --- | --- |
|  | Total = 109,406 | Predicted positive | Predicted negative |
| Actual condition | P = 78 | TP = 40 | FN = 38 |
|  | N = 97,754 | FP = 0 | TN = 97,754 |

P, Positive; N, Negative; TP, True Positive; FP, False Positive; FN, False Negative; TN, True Negative.

**Table S2. Prediction outcomes for ribotricer on *A. thaliana*.**

|  |  | Predicted outcomes | |
| --- | --- | --- | --- |
|  | Total = 109,406 | Predicted positive | Predicted negative |
| Actual condition | P = 78 | TP = 57 | FN = 21 |
|  | N = 97,754 | FP = 529 | TN = 97,225 |

P, Positive; N, Negative; TP, True Positive; FP, False Positive; FN, False Negative; TN, True Negative.

**Table S3. Prediction outcomes for RIBOSS on *H. sapiens*.**

|  |  | Predicted outcomes | |
| --- | --- | --- | --- |
|  | Total = 7,022 | Predicted positive | Predicted negative |
| Actual condition | P = 126 | TP = 39 | FN = 87 |
|  | N = 4,475 | FP = 12 | TN = 4,463 |

P, Positive; N, Negative; TP, True Positive; FP, False Positive; FN, False Negative; TN, True Negative.

**Table S4. Prediction outcomes for ribotricer on *H. sapiens*.**

|  |  | Predicted outcomes | |
| --- | --- | --- | --- |
|  | Total = 7,022 | Predicted positive | Predicted negative |
| Actual condition | P = 126 | TP = 53 | FN = 73 |
|  | N = 4,475 | FP = 109 | TN = 4366 |

P, Positive; N, Negative; TP, True Positive; FP, False Positive; FN, False Negative; TN, True Negative.

**Use of LLMs**

LLMs were used as grammar and spell checkers to improve the clarity of the main text and the GitHub README. LLMs were also used to identify inconsistencies in the text and code.
